# Supplementary material for: Effects of Trichoderma harzianum combined with Phanerochaete chrysosporium on lignin degradation and humification during chicken manure and rice husk composting
Source: Front Microbiol. 2025 Feb 28;16:1515931. doi: 10.3389/fmicb.2025.1515931 (PMC11906335; doi:10.3389/fmicb.2025.1515931)
Supplement: Supplementary file 1 [file Data_Sheet_1.zip › 6.Function/BFHS_CKF/FunGuild/l1/metastats/Saprotroph.CKF_BFHS.metastats.pdf]

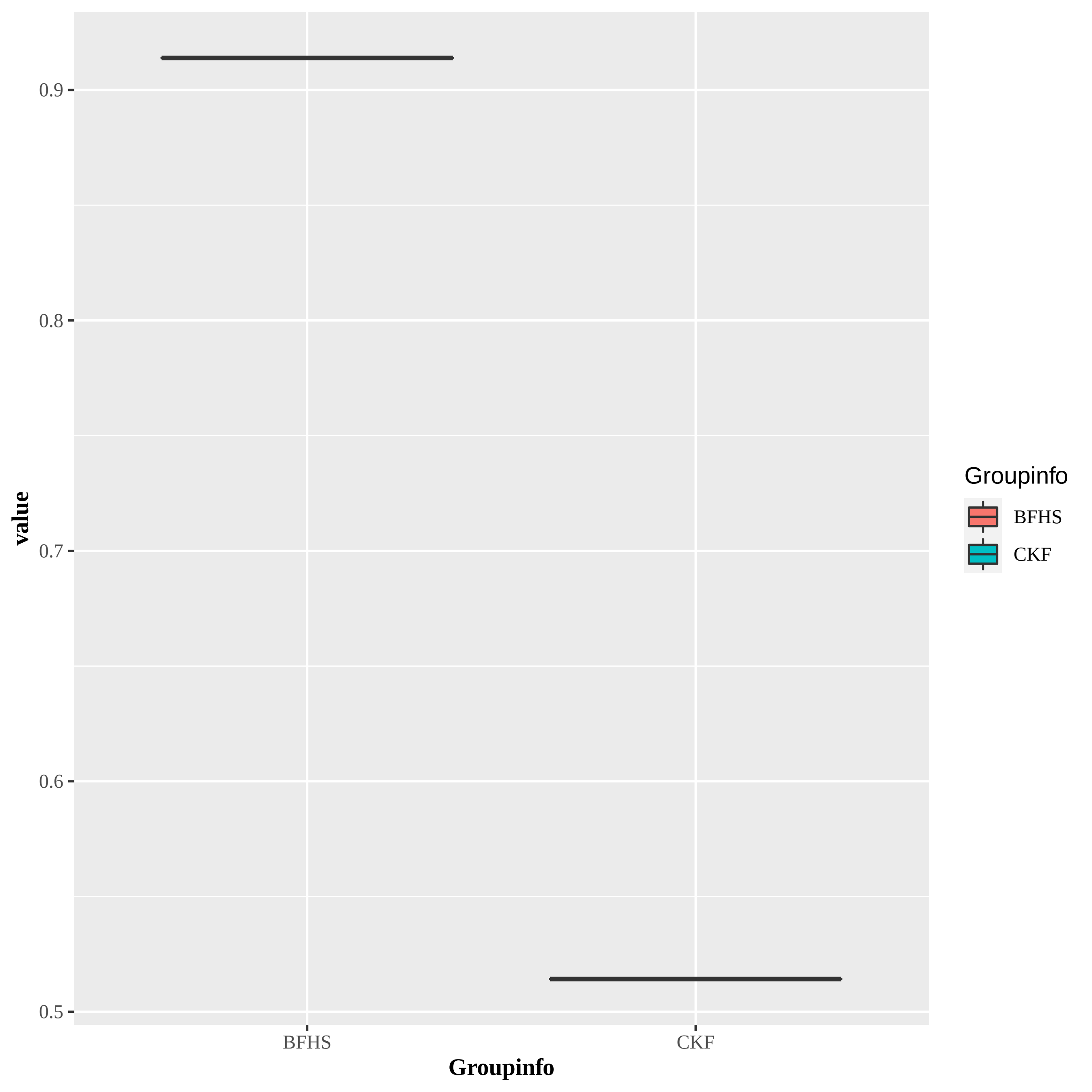

0.9
0.8
0.7
0.6
0.5
value
BFHS
CKF
Groupinfo
Groupinfo
BFHS
CKF
